# Inactivation and Environmental Stability of Zika Virus

**DOI:** 10.3201/eid2209.160664

**Published:** 2016-09

**Authors:** Janis A. Müller, Mirja Harms, Axel Schubert, Stephanie Jansen, Detlef Michel, Thomas Mertens, Jonas Schmidt-Chanasit, Jan Münch

**Affiliations:** Ulm University Medical Centre, Ulm, Germany (J.A. Müller, M. Harms, A. Schubert, D. Michel, T. Mertens, J. Münch);; Bernhard Nocht Institute for Tropical Medicine, World Health Organization Collaborating Centre for Arbovirus and Hemorrhagic Fever Reference and Research, Hamburg, Germany (S. Jansen, J. Schmidt-Chanasit);; German Centre for Infection Research partner sites, Hamburg-Luebeck-Borstel, Germany (S. Jansen, J. Schmidt-Chanasit)

**Keywords:** Zika virus, Flavivirus, inactivation, viruses, vector-borne infections

**To the Editor**: Zika virus is an emerging virus that has spread to most countries in Latin America and the Caribbean (*1*,*2*). It is transmitted by mosquitoes and through sexual intercourse (*3*). Most persons infected with Zika virus are asymptomatic or experience mild symptoms (*4*). However, in a pregnant woman, infection may cause severe pregnancy and birth complications, most notably microcephaly in children infected in utero (*5*–*7*). Zika virus infection might also be associated with an increased incidence of Guillain-Barré syndrome (*8*). Thus, the virus represents a threat to healthcare workers who manage infected patients or diagnostic samples and researchers who work with infectious virus in laboratories. 

Working with Zika virus, a Biosafety Level 2 (BSL-2) pathogen in the European Union, except for the United Kingdom (where it is BSL-3), requires specific safety precautions (*9*). No inactivation data specific for Zika virus are available (*9*); consequently, disinfection guidelines are based on protocols to inactivate other flaviviruses. To gain experimental evidence regarding inactivation and disinfection for Zika virus, we determined its susceptibility to various disinfectants and inactivation methods.

To test susceptibilities, we determined the 50% tissue cell infectious dose per milliliter (TCID_50_/mL) (*10**)* of the Zika virus MR766 strain (*1*) before and after the virus was exposed to disinfectants or other inactivation procedures (Technical Appendix). We then determined the effect of alcohol-based disinfectants on viral infectivity. Using Zika virus stock containing 2.5% fetal calf serum (FCS) mixed 3:10 (vol/vol) with indicated alcohols, we incubated the mixture for 1 minute and then used it for infection (Figure, panel A). All alcohols entirely inactivated the virus. Complete loss of infectivity was also observed after virus exposure to 1% hypochlorite (often used to inactivate virus in liquid wastes in BSL-2/3 laboratories), 2% paraformaldehyde (used to inactivate virus for subsequent flow cytometry), and 2% glutaraldehyde (often applied to fix virus for subsequent electron microscopy analysis) (Figure, panel A). Thus, routinely used disinfectants and inactivation procedures are sufficient to inactivate Zika virus in laboratory virus stocks. Next, we repeated these experiments in the presence of a high protein load using Zika virus preparations supplemented with FCS in increasing concentrations (10%, 40%, 90%), to mimic virus found in clinically relevant material. Again, all treatments entirely disrupted Zika virus infectivity (Figure, panel A).

**Figure:**
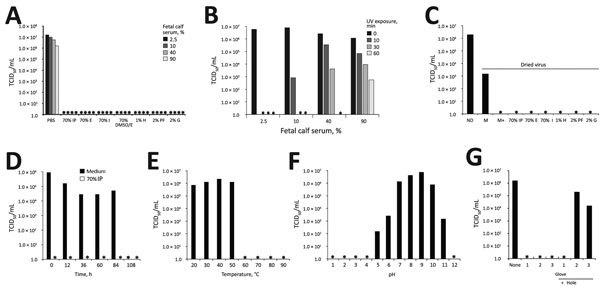
Inactivation and environmental stability of Zika virus. Asterisks (*) indicate lack of infection. A) Virus stocks containing 2.5%, 10%, 40%, or 90% fetal calf serum were incubated with alcohol-based disinfectants for 1 min. All disinfectants inactivated the virus. B) Virus stocks containing indicated concentrations of fetal calf serum were exposed to the ultraviolet (UV) light of a laminar flow hood. Higher concentrations of serum required more time to inactivate the virus. C) Virus stock was dried for 18 h and was then reconstituted in medium or the indicated disinfectants for 5 min or exposed to 10 min UV light before reconstitution. All disinfectants inactivated the virus. D) Virus was dried and incubated for indicated periods of time. Thereafter, dried virus was reconstituted in medium or 70% (vol/vol) isopropanol. Isopropanol inactivated the virus, but dried virus in medium remained infectious even after 84 h of incubation. E) Zika virus was incubated for 5 min at indicated temperatures. Temperatures >60°C inactivated the virus. F) Stocks were adjusted to indicated pH values and incubated for 10 min. pH levels <4 or >11 deactivated the virus. G) Finger tips of laboratory gloves were cut off, with or without introducing a hole by pinching with a needle, and put into medium. Glove tips were filled with virus stock and incubated for 90 min at room temperature. All gloves without needle holes were protective against transmission; 2 of 3 gloves with needle holes allowed virus transmission. For detailed experimental description, see Technical Appendix. DMSO, dimethyl sulfoxide; E, ethanol; G, glutaraldehyde; H, hypochlorite; I, incidin, IP, isopropanol; M, medium, M+, medium plus 10 min UV; ND, not dried; PBS, phosphate-buffered saline; PF, paraformaldehyde; TCID_50_, 50% tissue culture infective dose; UV, ultraviolet.

Ultraviolet (UV) radiation inactivates viruses by chemically modifying the genome. We exposed 200 μL of Zika virus preparations containing increasing concentrations of serum to UV light of a laminar flow for up to 60 minutes. Exposure for 10 minutes entirely inactivated Zika virus in the presence of 2.5% FCS serum; increasing concentrations of serum reduced the antiviral effects of UV light (Figure, panel B). When Zika virus containing 90% serum was exposed for 60 min to UV light, infectivity was reduced by 99.95%; however, some residual infectivity was detected (Figure, panel B).

Next, we evaluated environmental stability by drying 100 μL of Zika virus stock for 18 hours. Thereafter, dried virus was reconstituted in the same volume of medium or disinfectants. Endpoint titrations showed that the reconstituted virus remained infectious, although TCID_50_ was reduced by ≈3 orders of magnitude (Figure, panel C). All disinfectants and UV radiation entirely inactivated dried Zika virus (Figure, panel C). Additional experiments demonstrated that dried Zika virus remained infectious for >3 days (Figure, panel D) suggesting, for example, that dried droplets can be infectious, confirming that proper surface disinfection is essential.

We also assessed the environmental stability of Zika virus to heat and change in pH. The virus was stable at temperatures up to 50°C but lost all infectivity at temperatures of >60°C (Figure, panel E). Thus, virus-contaminated materials such as surgical instruments can be decontaminated by heat. We also found that Zika virus infectivity was highest after adjusting the stock to a pH of ≈9 (Figure, panel F). In contrast, adjusting Zika virus to pH 12 or to <pH 4 abrogated infectivity (Figure, panel F).

Finally, we analyzed whether gloves routinely used in BSL-2 laboratories protect against Zika virus. For this, we cut off fingertips of nitrile and latex gloves, filled tips with a Zika virus suspension, and placed them into cell culture plates containing medium. Virus-containing fingertips were inserted in such a way that diffusion would only occur if the virus passed through the nitrile/latex barrier. As a control, we made a hole of <1 mm in the fingertips. All 3 tested gloves prevented virus diffusion (Figure, panel G). However, if glove integrity was disrupted by a pin, the virus passed through in 2 of 3 cases (Figure, panel G).

We demonstrated that Zika virus is destroyed by classical disinfectants and inactivation methods and that nitrile and latex gloves are protective. We also showed that UV light of a laminar flow hood inactivates Zika virus, but particularly if the virus is in a protein-rich environment, the exposure time range should be in hours rather than in minutes. Although we expected that Zika virus would be inactivated by alcohol and disinfectants, we conducted a thorough experimental verification to exclude uncertainties surrounding work with this emerging pathogen.

Technical Appendix Methods of testing susceptibility of Zika virus to various disinfectants and inactivation methods.
