# Supplementary material for: Inactivation and Environmental Stability of Zika Virus
Source: Emerg Infect Dis. 2016 Sep;22(9):1685–7. doi: 10.3201/eid2209.160664 (PMC4994368; doi:10.3201/eid2209.160664)
Supplement: Technical Appendix — Methods of testing susceptibility of Zika virus to various disinfectants and inactivation methods. [file 16-0664-Techapp-s1.pdf]

Article DOI: http://dx.doi.org/10.3201/eid2209.160664

Inactivation and Environmental Stability of Zika Virus

Technical Appendix

Cells, Viruses, and Reagents

Vero E6 cells, used for propagation and infection of Zika virus (ZIKV), were grown in Dulbecco modified Eagle medium (DMEM) supplemented with 2.5% inactivated fetal calf serum (FCS), 200 mM L-glutamine, 100 units/mL penicillin, 100 μg/mL streptomycin, 1 mM sodium pyruvate, and nonessential amino acids (Sigma #M7145) at 37°C in a 5% CO_2–humidified incubator. ZIKV strain MR 766 was isolated in 1947 from a sentinel rhesus macaque.

Generation of ZIKV Stock

We infected 70% confluent Vero E6 cells in T175 cell culture flasks with ZIKV MR 766 for 2 h in 5 mL medium. Subsequently, 40 mL fresh medium was added, and the cells were cultured. Cytopathic effect (CPE) was monitored by light microscopy, and virus was harvested when 70% of the cells detached due to CPE. Supernatant was taken, centrifuged for 3 min at 1,300 rpm, before virus stocks were aliquoted and stored at –80 °C. The ZIKV stock used throughout this study had an infectious titer of 1.6×10^7 $TCID_{50}$/mL and a genome copy number of $\approx 1 \times 10^{10}$ /mL as assessed by quantitative reverse transcription PCR (RT-PCR) (RealStar Zika Virus RT-PCR Kit; Altona Diagnostics, Hamburg, Germany).

Effect of Alcohols, Disinfectants, and Other Chemicals on ZIKV Infection

To determine ZIKV sensitivity to disinfectants with different protein loads, we adjusted the virus stock to 2.5%, 10%, 40%, and 90% (v/v) FCS. Virus was then mixed 3/7 (v/v) with H_2O, isopropanol (VWR International #20842.330), ethanol (VWR International #20821.330), incidin (ECOLAB Healthcare #3021780), DMSO/ethanol (Merck #1029521000), 1% hypochlorite (PanReac AppliChem # 213322), and 2% paraformaldehyde (Merck #1040051000), and glutaraldehyde (PLANO #R1012) (v/v). Following a 1-min incubation, samples were serially diluted and used to inoculate VeroE6 cells. MTT assays in absence of virus were

performed at all times to check for cytotoxicity of the chemicals after dilution and inoculation of cells.

Effect of Ultraviolet (UV) Light on ZIKV Infection

To determine ZIKV sensitivity to UV light, we placed 100 μL of ZIKV stocks, supplemented with different concentrations of serum, into a reagent reservoir and exposed to bench UV light (HNS Puritec 15W G13 OFR. 200–260 nm, Osram) for indicated times. After irradiation samples were serially diluted, $TCID_{50}$ was determined.

Effect on Disinfectants of Dried ZIKV Solution

For ZIKV stability upon drying to be determined, 200 μL of virus stock solution was dried on a 24 cell culture plate for 18 h. The dried samples were then reconstituted in an equal volume of disinfectant or phosphate-buffered saline. One samples was UV-irradiated before reconstitution. Samples were then serially diluted, and the $TCID_{50}$ was determined.

Stability of ZIKV Infectivity over Time

To analyze virus infectivity over time, we mixed virus stock with 70% medium or isopropanol (v/v), and incubated the mixture at 37°C. At indicated time points, samples were taken and stored at –80 °C until they were thawed in parallel and analyzed as described.

Temperature sensitivity of ZIKV

The virus stock was incubated in 1.5-mL reaction tubes at indicated temperatures for 5 min. $TCID_{50}$ was then determined.

pH Sensitivity of ZIKV

Virus was mixed 1:1 (v/v) with medium to result in the indicated pH values during a 10-min room temperature incubation. Medium with different pH was generated using a pH-meter and confirmed by pH paper after mixing with virus. Samples were then titrated and used for inoculation.

Glove Protection against ZIKV

For this, we cut off the fingertips of nitrile gloves (Sempercare nitrile skin², Sempermed; and Peha-soft nitrile fino, Hartmann) and latex gloves (Sempercare premium latex, Sempermed), filled the tips with 100 μL of ZIKV suspension, and placed them into 24-well plates containing 500 μL medium. The ZIKV-containing fingertips were placed in a way that diffusion can only

occur when the virus passes through the nitrile/latex barrier. As a control, we inserted a hole of <1 mm in 1 fingertip by pinching it with a needle prior to loading with ZIKV. After 60 min, gloves were removed, and the medium below was serially diluted to determine $TCID_{50}$.

$TCID_{50}$ Endpoint Titration

We seeded 6,000 Vero E6 cells per well in 96-well plates in 100 μL medium and incubated the plates overnight. The next day, medium was taken and replaced with 180 μL fresh medium. For endpoint $TCID_{50}$ determination, ZIKV samples were serially diluted 10-fold, and 20 μL of each dilution was used for inoculation of Vero E6 cells. This endpoint titration resulted in final ZIKV dilutions of 10^1–10^9 fold on the cells in triplicates. Cells were then incubated and monitored for virus induced CPEs and plaque formation. $TCID_{50}$/mL was calculated according to Reed and Muench. The used ZIKV stock in this study had an infectious dose of 1.6×10^7 $TCID_{50}$/mL.
